# Male patients’ preferences for opioid use treatment programs

**DOI:** 10.1186/s12888-023-04939-x

**Published:** 2023-06-16

**Authors:** Mostafa Amini-Rarani, Maryam Moeeni, Koen Ponnet

**Affiliations:** 1 Health Management and Economics Research Center, Isfahan University of Medical Sciences, Isfahan, Iran; 2 Social Determinants of Health Research Center, Isfahan University of Medical Sciences, Isfahan, Iran; 3 Faculty of social sciences, imec-mict-ghent university, Ghent, Belgium

**Keywords:** Opioid use disorder, Patient preferences, Opioid substitution treatments, Qualitative analysis, Iran

## Abstract

**Background:**

A patient-centered approach to the treatment of substance use is helpful in achieving positive treatment outcomes. This study aimed to explore male patients’ preferences for opioid use treatments.

**Methods:**

A qualitative study was conducted in Isfahan, a city in the center of Iran. The study sample included 64 male participants who had started treatment for opioid use disorder (OUD). Using a purposive maximum variation sampling procedure, seven treatment centers were selected as interview venues. The semi-structured face-to-face interviews were conducted in a private room in the selected centers. A hybrid inductive/deductive approach was used to thematize the interview transcripts.

**Results:**

A total of three themes and 13 subthemes on opioid treatment preferences were identified: treatment concerns (anonymity, social stigma, fear of treatment distress, and family concerns), treatment attributes (treatment cost, location of the treatment center, treatment period, frequency of attendance, informed treatment, and treatment personnel), and treatment type (maintenance or abstinence and residential and community treatments). The study showed that all treatment programs were perceived to have their own strengths and weaknesses.

**Conclusions:**

The results showed that patients with OUD carefully compare the positive and negative aspects of existing treatment programs, and they consider a treatment program to be a package of favorable and non-favorable qualities. The identified themes could inform policymakers about the treatment preferences of male patients and provide an opportunity to promote better treatment options for OUD.

**Supplementary Information:**

The online version contains supplementary material available at 10.1186/s12888-023-04939-x.

## Background

Opioid use disorder (OUD) is a public health concern and a social problem that affects the entire world [1]. Because OUD is characterized by a chronic relapsing nature, helping people recover from opioid use is very difficult [2–4]. Iran has one of the highest rates of opioid use in the world, and opioid use has consistently been a major problem. Patients with OUD constitute the highest proportion of people seeking therapies for drug use in Iran [5–7].

Therefore, the Iranian healthcare system began introducing internationally approved treatments for OUD patients in the second half of the 1990s [2, 8]. Thus, a well-developed and wide-ranging number of opioid treatment programs have been implemented with the support of the government, private sector, and non-governmental organizations [2, 8, 9]. The major programs apply one or more of the following strategies that may diverge or converge: opioid substitution therapy with methadone, buprenorphine, tincture of opium, and others; medical detoxification and other short-time pharmacological procedures; abstinence therapies; counseling and psychological and/or psychiatric services; residential rehabilitation and inpatient/outpatient therapies; and individual/self-help group therapies [2, 8].

Previous studies have shown the overall positive benefits of the programs provided in Iran [5–7, 10–15]. Because the available OUD treatment programs include different settings, supporting services, medications, and treatment goals [4, 16, 17], voluntary patients can choose among numerous treatment options, such as inpatient or residential versus outpatient treatment options, substitution therapies versus drug-free rehabilitation, short-run versus long-run therapies, individual versus group therapies, or public versus private centers [18, 19]. Providing a range of options can help patients find a more convenient treatment program based on their specific needs and preferences. Research evidence supports the positive role that patient-centered practice plays in healthcare outcomes and patient satisfaction [20–23]. Thus, existing treatment guidelines recommend that clinicians and treatment providers actively engage patients in the treatment process [23].

Despite these improvements, the country is still reporting unmet needs for opioid treatment [4, 5]. As a result, the majority of opioid use patients are not enrolled in treatment services. Moreover, a large number of patients who enroll in a treatment program drop out before program completion and relapse, or they switch frequently from one treatment to another [4, 24]. Despite the overwhelming amount of research on OUD treatment strategies [25–36], more scholarly attempts are needed to provide treatment programs that consider the preferences of a wide range of patients. Thus, the present study aims to contribute to the literature by exploring the perspectives of male patients regarding their preferred therapy options for OUD. Such knowledge could help public policymakers, healthcare system administrators, and treatment providers understand which aspects of treatment services best meet OUD patients’ preferences. Furthermore, it could help in designing a more appropriate treatment program that not only encourages more patients with OUD to undergo treatment procedures but also helps patients with OUD who are already enrolled in a treatment program to complete the therapy regimen.

## Methods

### Setting

A qualitative study was conducted in Isfahan, a city located in the center of Iran. The study sample included OUD patients who had already started a procedure to treat OUD. The first inclusion criterion was being male. The reason for this criterion is that the prevalence of drug use disorders among Iranian males is much higher than that among females. Furthermore, drug use behaviors, especially the process of seeking treatment for drug use disorders, in Iran are very different between males and females. The other patient inclusion criteria were continuous problematic opioid use for at least one year prior to the treatment start date, more than 18 years of age at the treatment start date, not being in the first week of treatment, and voluntary enrollment in the treatment program.

Several types of legal OUD treatment providers are available in Isfahan, such as public and private methadone maintenance treatment (MMT) centers, residential treatment centers (usually called camps), drop-in centers (DICs), and private inpatient treatment centers. Among these, MMT centers and camps are the most frequented. MMT is a prolonged outpatient treatment that provides OUD patients with medications prescribed by a physician for a long period of time. While most MMT centers are private, a few public centers also offer MMT [19]. Camps run by former drug users provide patients with residential but non-healthcare services (i.e., abstinence treatments) for a short period of time [4]. DICs are government-funded centers that provide harm reduction services for socially marginalized people with problematic substance use disorders. These patients can access medications and visits with physicians, which help them abstain from drug use [4]. Inpatient treatment centers are private clinics that offer assistance with opioid withdrawal using different medications, including methadone [4].

Using a purposive maximum variation sampling procedure, we selected seven sites as interview venues: two public MMT centers, two private MMT centers, two camps, a DIC, and a private inpatient treatment center. Note that although DICs are primarily harm reduction centers for drug users, they also occasionally offer some treatment services to those seeking therapies for drug use. Purposive sampling with a maximum variation approach was utilized at each site to determine possible sample variance in terms of age, education, marital status, and employment status in relation to each treatment procedure (more information can be found in Additional File 1). The respondents who met the inclusion criteria were identified through the sites’ staff referrals.

Semi-structured face-to-face interviews were conducted in a private room at the selected sites. Based on the available literature, the research expertise of the second author, and several informal conversations with patients about their ideal treatments, an interview guide was developed. The process of interview guide development involved five steps [37]:

1) *Identify the requisites for using semi-structured interviews*. Because opioid users had a low level of awareness of the subject, treatment-seeking issues were socially and emotionally sensitive topics, and thus, opioid users were not used to talking about them. Therefore, the semi-structured interview method was considered suitable for this study.

2) *Review previous knowledge*. The aims of the literature review were to gain a comprehensive understanding of opioid use treatment qualities and to create a conceptual basis for the initial codes and themes.

3) *Formulate the preliminary interview guide*. An interview guide was formulated as a list of questions based on previous knowledge.

4) *Pilot test the interview guide*. To confirm the coverage and relevance of the content of the preliminary interview guide and to detect any possible need to reformulate the questions, internal testing was conducted. For this, one author (MAR) assumed the role of the participant and was interviewed by another researcher (MM). Consequently, the research team removed ambiguities and inappropriate leading questions and reordered the questions.

5) *Present the complete interview guide*. In this step, a clear, finished, and logical interview guide for data collection was produced.

The second author conducted the interviews. The interviews started with the interviewer introducing herself, explaining her interests in the research topic, and describing the aim of the research project. Then, socio-demographic questions, questions about the types of opioids the respondents used, and the respondents’ drug use history were asked. These questions were followed by a general open-ended question: “What would be the characteristics of an ideal treatment option from your point of view?” To extract in-depth personal narratives and probe the topic, the interviewer also asked the patients about treatment costs; medications used; family attitude toward the treatment; anonymity, accessibility, and location of the treatment center; expected treatment duration; residential/nonresidential care; healthcare facilities associated with the treatment centers; patient–provider relationships; and informed/uninformed treatment procedure (the interview guide can be found in Additional File 2). Some field notes were taken by the interviewer.

Three patients dropped out of the interviews because they faced physical pain or anxiety. One patient stopped the interview without providing any special reported reason. Data gathering continued until the saturation point. This point was reached when the analysis and comparison of interview contents needed no new data. All interviews conducted in Persian. They lasted 13–84 min and were audio-recorded and transcribed verbatim. The data collection and analysis took place from January 2018 to March 2019.

### Data analysis

The analyses were based on the interview data provided by 64 participants, of which 34 occurred in MMT centers, 20 in camps, six in a DIC, and four in a private inpatient treatment center. A hybrid inductive/deductive approach was used to thematize the interview transcripts [38]. The interviews were reviewed several times to infer a list of inductive themes (for examining emerging codes and data-driven themes from the raw data). Simultaneously, deductive themes (a priori templates of codes derived from the literature review on opioid treatment qualities) were identified directly.

The transcripts were analyzed by two principal investigators (MAR and MM), and the interview data were coded using MAXQDA Analytics Pro 2020 (Release 20.0.8, VERBI GmbH Berlin). Thereafter, in a meeting, all inconsistencies were discussed and resolved by the researchers. The output was a list of elicited themes outlining the conditions of the OUD patients’ preferred treatment procedures. The researchers paid particular attention to the feasibility, applicability, and comprehensiveness of the interview guide, which can improve its transferability in qualitative studies. Furthermore, to prevent interviewer biases, the researchers never expressed their personal values to the interviewees.

## Results

The socio-demographic and therapeutic characteristics of the participants are shown in Table 1. The participants were aged 37 years old, on average, and had been living with OUD for more than 12 years. The majority of the respondents were married, educated, and undergoing treatment in private MMT centers. Patients would use at least one type of opioid. They reported opium, heroin, Asian crack (a heroin-based substance), and opium juice as their main drugs of choice.

**Table 1:** , Descriptive characteristics of the participants

Variables			Mean (SD)	Frequency (%)
Age			37.1 (9.7)	
Addiction duration (year)			12.3 (7.2)	
Education level	Academic			9 (14.1%)
	High school			24 (37.5%)
	Secondary			16 (25%)
	Primary			13 (20.3%)
	Illiterate			2 (3.1%)
Marital status	Married			33 (51.6%)
	Single			29 (45.3%)
	Divorced			2 (3.1%)
Treatment centre type	MMT	Public		13 (20.3%)
		Private		21 (32.8%)
	Camp	Public		10 (15.6%)
		Private		10 (15.6%)
	DIC			6 (9.4%)
	Private inpatient centre			4 (6.3%)

Table 2 describes three themes and 13 subthemes regarding the patients’ preferences for opioid use treatment programs. These themes, subthemes, and issues are described in greater detail later in this study.

**Table 2:** , Themes, sub-themes and codes related to the preferred opioid use disorder treatment program

Theme	Subtheme	Issue
treatment concerns	*Anonymity*	- Relatives and acquaintances - Closed family members - Intimate friends - Unacquainted individuals
	Social stigma	- Indignity - Stigmatize opioid users - Harm the reputation of opioid users - Embarrassment of opioid users
	Fear of treatment distress	- Fear of hangover - Fear of painfulness - Fear of insomnia - Fear of nervousness and upsetting - Fear of relapse - Fear of physical disorder - Fear of mental disorder - Fear of adverse quality of life
	Family concerns	- Family consent - Family emotional support
Treatment attributes	Treatment cost	- High cost of treatment - Unaffordability of cost - Having no money
	Location of the treatment center	- Within neighborhood - Out of neighborhood - Within city - Suburb
	Treatment period	- Shorten the duration of treatment - Flexibility of the length of treatment
	Frequency of attendances	- Frequency of attendances for receiving methadone - Attending in consultation meetings
	Informed treatment	- Medical specialists - Mass media - Books and articles - Peer drug-free patients
	Treatment personnel	- Personnel-patient communication - Personnel’s specialty
Treatment type	Maintenance or abstinence	- Easiness of treatment - Painlessness - Doing routine affairs during treatment process - Retaining job and doing work together with continuing treatment process - Physical side effects - Extended treatment - Drug replacement - Dual addiction - Impossibility of withdrawing methadone
	Residential or not	- Painfulness - Unsuitable atmosphere - Restricted freedom - Forced to withdraw - Limited visit to the family and communication - Make up the indignation - Find new addicted mates - Keep away from opioids and addicted peers
	Community treatment or not	- Empathy - Providing real to avoid withdrawal syndrome - Sharing hardships and sufferings - Purification of the mind from the temptations of opioid use - Supplement for other treatments - Encouragement to stay opioid free

### Treatment concerns

#### Anonymity

According to the participants, whether they were treated confidentially depended on their social environment (e.g., the presence of individuals who supported them and their relationships with them). Although they had fewer concerns about whether close family members were aware of their treatment process, they had different views where non-family members were involved. That is, while they preferred that their treatment be kept secret from non-family members, this was not the case where their close family members were concerned:

*I don’t want to talk to strangers; I don’t want anyone to know what I’m doing. In my opinion, when someone understands my problem, they never trust me. For example, whenever I get angry about something else, other people think that I have a problem and that I use drugs ... So, it is better that no one knows what problem I had once.* (Participant 54)

*I would like my family to know that I am in recovery because they will be happy and help me. But I don’t want others to know that I was once an addict.* (Participant 41)

However, this perception differed when it came to opioid users’ friends, depending on whether these friends were also opioid users. Because opioid users wanted to prove themselves and win their non-user friends’ confidence and acceptance, they preferred to allow their non-user friends to know about their treatment. As for their opioid user friends, some patients preferred not to let them know about their treatment. OUD patients may be convinced by their peers to relapse, and this may lead to interference in the treatment process, so some respondents were hesitant to inform their peers. In this regard, one of the participants expressed the following:

*I’m here, and my friends don’t know about it. The more that they don’t know, the better it is for me. If I contact them, it’s the same old story. The last time I went to the camp and then withdrew, they understood, and they kept coming to me and persuading me that they had good drugs and that they’re new ones, and ... come on, I’ve always been tempted ... Although I tried to stay away from my close friends, I’d always dreamed of drugs, so I relapsed.* (Participant 27)

However, some other participants claimed that letting their opioid user friends know about their treatment motivated them to complete their therapy. In hearing that their peers had been able to quit, they felt hopeful that they could achieve the same goal.

#### Social stigma

OUD patients may be identified and stigmatized when seeking treatment. They feel embarrassed, as though they have lost their dignity and destroyed their reputation because of the harmful social and cultural stigmas associated with individuals who suffer from OUD. Therefore, these patients preferred a treatment program that did not have any social stigma associated with it.

The participants expressed that as a result of seeking treatment in the treatment centers, they were recognized as opioid users and experienced the social and cultural stigmas associated with OUD. One of the participants described this situation as follows:

*The addict is known as the Village Pump. Now, many individuals often don’t consider an addict to be a patient. Write the word “addict” and then write whatever you want; that is, they call an addict everything. Parasites, robbers, and philanderers—they intend to ascribe these negative views to an addict.* (Participant 6)

*You see, addiction has its own consequences. It is enough for one to be known as an addict. They attach any stigma to him. Unfortunately, it is common for a person who uses a drug to have no credibility. People easily stigmatize him … They treat him like trash.* (Participant 42)

#### Fear of treatment distress

Patients may be afraid to start a treatment program because they perceive it to be dangerous, painful, or harmful. A treatment process might be preferred if it helps patients overcome treatment apprehension. Patients seemed to prefer a treatment program that could reduce their fear of treatment-related side effects and distress. Unpleasant emotions or feelings caused by opioid treatments were the worry of many opioid users. Thus, many patients with OUD preferred treatment programs that address the fear of treatment programs. For example, one participant said the following:

*Fear of the side effects of a treatment, yeah, everybody’s afraid of hangovers, physical pain, nervousness, mental illness, clumsiness, and insomnia. It seems impossible for us to be free from these .... They are scary, and they can cause a person not to participate in a treatment program ... Okay, these fears need to be minimized.* (Participant 50)

Furthermore, OUD patients who join treatment programs generally suffer from severe withdrawal symptoms that scare them, including physical and mental effects (e.g., aggression, insomnia, anorexia, headache, total body pain, impotency, anxiety, hysteria, and fainting) and undesirable effects on their quality of life (less intimate communication, less leisure time, fewer recreational activities, and less happiness). Therefore, many participants preferred treatment centers that could manage the fear of withdrawal effects. The interviewees shared that opioid treatment is often accompanied by physical and mental disorders. This negatively affects their quality of life. However, some treatment centers do not take the side effects of treatments into account, so many patients complained about such treatment centers. Two interviewees in a rehabilitation camp reported the following:

*You see, withdrawal syndrome during treatment is really bothersome because the person who uses drugs no longer has a normal body. As the saying goes, the body’s systems completely disintegrate ... like a glass that breaks, and then you want to stick the parts together again. I’ve been using drugs for 11 years, so I’ve never felt like I’m going to quit. But just a couple of days after I quit, my hair turned white… I lost one of my teeth, which did not happen to me when I used drugs. Not taking opioids really leaves the body sick. Personally, I’m not in agreement with that.* (Participant 32)

*In a camp like this, when a poor guy has severe stomach pain, he always moans that his stomach aches, he’s in a really bad mood, and so on. They tell him he’s taking drugs. You then endure the pain. He would squirm in pain before nightfall, before he dies, but if he had been rushed to the hospital, something would have been done to save him.* (Participant 57)

#### Family concerns

Family consent regarding the treatment method and family emotional support during and after the treatment were among the main concerns of the respondents when it came to an acceptable treatment process. The participants shared that when family members were aware of and agreed to the treatment decision, they helped support the treatment process (emotionally and even financially). Particularly in the case of married opioid users, the loving care and understanding of their spouses were crucial factors in seeking treatment and keeping their spouses away from opioid use. The presence of OUD patients’ parents, spouses, and children in the treatment centers helped patients feel valued, respected, loved, and protected; their frustration induced by addiction diminished as a result.

Regarding residential treatment, visits from family were also seen as desirable: *“Definitely, meeting with my family is necessary. They’re really important in supporting me. I’m here to make my family proud and satisfied”* (Participant 32).

### Treatment attributes

#### Treatment costs

The cost of treatment is one of the critical attributes affecting the appeal of a treatment option. The majority of respondents expressed that they did not seek treatment simply because they could not afford it or because the treatment was expensive: *“I haven’t always had the money; I haven’t gone to the treatment program because of money-related problems; that is, I don’t have the funds to participate in the treatment program”* (Participant 62).

Overall, many OUD patients indicated that they could not pay for treatment and/or faced exorbitant costs and poverty induced by treatment, mostly because they were unemployed or underemployed. A participant being treated at the public MMT center said the following:

*After all, someone who’s addicted doesn’t have much money, and they’re not rich. Rich opioid consumers can be rare—1 out of 100. It’s generally difficult for a user to pay because many users don’t have a decent job or are unable to work. Most addicts, even if they have a healthy body, need to stay at home.* (Participant 14(

#### Location of the treatment center

The location of the treatment center was also important to the participants. Some participants preferred treatment centers located in their neighborhoods because of their easy access (i.e., within the vicinity of their homes or work):

*It’s close to my workplace and my home. It was easy for me to visit the doctor. If the treatment center were far away, then I would be worried about how to visit the doctor, especially in cold, rainy, and snowy weather. Now, I can come here from that side of the street on foot, visit the doctor, and return home quickly.* (Participant 47)

Other participants preferred treatment centers located outside their neighborhoods because they could keep their visits discreet, and they were far from their peers and drug hangout locations: “*Well, it is better here that it is far from where I live because no one sees or understands … I know a camp in our own city, but I don’t go there”* (Participant 3).

In terms of city or suburban treatment center locations, there are several pros and cons. A respondent being treated at a private camp said the following:

*There are a number of benefits to having a treatment center in the suburbs and a number of other benefits to having it within the city. If the center were in the city, families can come and visit more easily because it’s closer, or when one of the patients gets sick, it’s easier to access healthcare, and doctors can help the patient quickly; so, it’s good. On the other hand, if the center were far away and outside the city, it’s better because some addicts have conflicts with one another, make noise, engage in self-harm, and so on. Also, if the center were in the city, it wouldn’t have a good influence. Children can see patients, and they may be affected. Clients may become aggressive and lose their temper during the treatment process. They might want to run away and bang on doors and walls, fight, and shout continuously; if the center were in the city, these conditions may result in the annoyance of neighbors.* (Participant 30)

#### Treatment period

The time allocated to the treatment program was another characteristic that affected treatment preferences. Notwithstanding differences in treatment duration, the respondents unanimously complained about prolonged treatment and believed that such treatments cause exhaustion, adverse effects, and high costs. In the case of methadone treatment, the participants were of the view that an extended treatment duration would not only increase the adverse effects of methadone on the body but also create a new drug use disorder. Prolonged treatment is also seen as unhelpful in residential treatment. During residential treatment, patients do not have access to drugs and are unable to use them, so they may pretend to comply with the treatment but relapse as soon as they can access the drug.

Regarding the flexibility of treatment duration, given that the experience of drug use is unique to each individual and drug use treatment is unique and based on the physical and mental capacity of each individual, the duration of treatment was perceived as follows:

*You know, the treatment period cannot be fixed to a certain amount of time. It depends, in my opinion, on the person and the physician in the center. The physician supervising an addict knows the best amount of time needed to treat them. I mean, two or three months is enough for me right now, but it’s not the same for others. There are some people, for example, who can be successfully treated for opioid use in two to three months, whereas some individuals, like me, need more time. You’ve got to work on the mind, the body, and the way of thinking. All this has to be done, and you also have to work with the family. Well, these are all time consuming. That’s why I don’t think there’s a set time for treatment duration.* (Participant 40)

#### Frequency of attendance

The frequency of methadone administration and consultation meetings, especially for maintenance treatment patients, was identified by the participants as another important attribute: *“The sooner I get rid of it, the better. After all, methadone is a drug that replaces drugs; the sooner I quit it, the better. I think it’s better for my work and my personal life”* (Participant 12).

The employed OUD patients tended to limit their attendance to avoid interruptions in their work lives. Other patients preferred frequent attendance because they felt that they were more involved in the treatment process when they visited the center and sought consultations often: *“My opinion is that I’d better quit drug use as gradually as I entered it. I’d better quit it the same way—as gradually as I reached the peak of drug use. To do this, I should get more and more involved with treatment”* (Participant 54).

#### Informed treatment

Another extracted subtheme was the preference of OUD patients for certain information related to various types of opioid treatments and for information during the treatment process. Many participants preferred informed treatment via medical specialists, mass media, books, articles, and even peers who were drug free and expressed that being uninformed about opioid treatment was a main obstacle to treatment-seeking behavior. An interviewee noted the following:

*I think it’s a lot easier when I hear about treatment. When you know how to deal with your fears—for example, when you learn something about opioid treatment—then you know what to do or what not to do. If treatment strategies have been introduced, it’s really important [to know about them] because many individuals just don’t know, and a lack of understanding leads to fear. But once they’re aware, it’s a lot better, which in turn helps lessen the fear, and they seek advice.* (Participant 49)

#### Treatment personnel

The interviewees mentioned a series of characteristics of treatment centers. Particularly, they discussed treatment personnel in terms of personnel–patient communication and personnel specialty. Regarding personnel communication, one of the interviewees commented the following:

*One thing to say is that addicts are sensitive individuals, and they’re searching for excuses. Because they’re [sensitive] individuals and delicate, they’re very happy with small things, and they also easily become very upset with something small. All these make them look for excuses too early and do not follow the treatment. I saw in the clinic that there was a young boy, and he was the only son; his family took great care of him. The clinic counselor told him that because he’s an only child, he’s babyish, niminy-piminy, and… it made him really sad; that’s why he left the clinic.* (Participant 57)

The interviewees also reported that the treatment centers should collaborate with other physicians, who are specialized when some side effects occur, including dentistry, psychiatry, neurology, gastroenterology, cardiology, and orthopedics.

### Treatment type

#### Maintenance or abstinence

Opioid maintenance treatment involves medications, such as methadone, buprenorphine, and opioid tincture. Opioid abstinence treatments do not utilize any medications or opioid replacement therapies for OUDs. Aside from a few participants who wished to seek maintenance treatment for their convenience and painlessness, the respondents continued their everyday lives while undergoing opioid treatment; most did not prefer maintenance treatment: *“I’d love to be treated in such a way that no other medications are used”* (Participant 51) and *“It’s much better to withdraw without drugs”* (Participant 49).

Others highlighted the adverse effects of medications, particularly methadone, including liver damage, a prolonged treatment process, drug substitution, dual substance use disorder, and the impossibility of withdrawing and stopping methadone. One participant who had been treated with methadone stated the following:

*I was undergoing methadone therapy, but it didn’t help at all. I was just getting methadone and taking drugs simultaneously. Methadone didn’t have any effect on me. I tried to use methadone again, but withdrawal was not easy; it was worse than with other drugs. Methadone treatment, in my view, makes no sense at all because methadone use can never be completely stopped. I didn’t want to take methadone; I couldn’t eat any more. I used methadone and put my drug next to it, so I took two drugs. I suffered from using two drugs.* (Participant 33)

#### Residential or not

In residential treatment, patients with OUD receive treatment services in residential facilities, such as camps and inpatient centers. However, there are patients who receive treatment services (e.g., medications and consultations) from nonresidential facilities, including outpatient centers (office-based treatments) and DICs. Generally, the participants did not have a good experience with residential treatment. They reported that the only benefit of residential treatment was being kept away from their old environment, opioids, and their addicted peers:

*Addicts suffer from serious problems. When we’re exposed to the extramural environment, the environment where we took drugs, the person who purchased the drugs, or the location where we used the drugs, all of it makes us think again—let’s relapse. Ah, we’re far from all that in the camp.* (Participant 55)

However, most of the participants mentioned deficiencies in residential treatment, including pain, an unsuitable environment, a lack of freedom, limitations in family visits, indignation, and the search for new addicted mates. In this respect, several participants with experience with residential treatment noted the following:

*The cost of the camp is much lower than here [private methadone treatment center], but the camp is a place with individuals who are likely to be offenders. Another issue is the immoral conduct that’s taking place in camps, which is not permitted and is unethical. I don’t want to go back to camp. I need to move to a place without such issues. In many camps, when addicts are forced to be treated, they become frustrated. As soon as they leave the camp, they try to make up for their anger, which in turn results in a relapse.* (Participant 58)

*In a camp, addicts make new friends, talk about the pleasures of taking new drugs, and are tempted to take some drugs after discharge.* (Participant 40)

*The camp is not a good place; you don’t have freedom, like a prisoner without visitation rights. It’s no different from being a bird with no wings.* (Participant 5)

#### Community treatment or not

In community treatment, groups of opioid users provide mutual support to one another in self-help groups and are involved in counselling/talk therapy programs. Most participants preferred collective treatment for a couple of reasons. Empathy has been described as a key feature of community treatment. In counseling meetings, OUD patients can understand the feelings of other patients. Moreover, staff members might have experienced drug use disorders themselves or could put themselves in OUD patients’ shoes.

Regarding empathy—the ability to put oneself in the place of another and understand someone else’s feelings—a participant said the following:

*Well, in meetings, you can meet your peers. Other people express the pain and sorrow they’ve experienced in life. I might be ashamed to talk about those pains, but I’ve experienced the exact same pain before, so I listen to the solutions, empathetic feelings, and compassion that other members bring. The members might be talking to another guy, but it’s as though they were talking to me. I am relaxed by the time I leave the meeting because I heard others. I received understanding and learned about my peers’ experiences, and I sought the right solutions. Consulting meetings are really successful.* (Participant 55)

Some participants reported that specific solutions to avoid withdrawal syndrome and cope with pain are provided through community treatment. Because psychological and social factors are the main drivers of relapse, opioid users need to be free from temptation during community treatment. As members acknowledge the importance of successful and long-term treatment, OUD patients are encouraged to both pursue treatment and remain opioid free for a long time. Finally, several participants favored community-based treatment as a supplementary therapy to other maintenance and residential treatments.

## Discussion

Identifying the treatment preferences of OUD patients can guide clinicians and treatment providers in providing the best OUD treatment services. This study examined preferred treatment options from the perspective of OUD patients. To the best of our knowledge, no previous study has focused on the treatment preferences of male patients in Iran. This study identified three aspects of OUD patients’ treatment preferences that played a key role in their search for treatment and can inform a number of policy implications. Policymakers and health providers need to consider treatment concerns (anonymity, social stigma, fear of treatment distress, and family concerns), treatment attributes (treatment cost, location of the treatment center, treatment period, frequency of attendance, informed treatment, and treatment personnel), and possible treatment types (maintenance or abstinence and residential and community treatments) when proposing or offering an OUD treatment package.

The majority of respondents reported that a favorable treatment program should take their treatment concerns into account. The respondents suggested the need for approaches and facilities that prevent stigma, fear, and distress, enable anonymity, and help resolve family concerns. Our participants felt that these features would help them enroll in and complete a treatment program.

The finding that many OUD patients preferred anonymous treatment is consistent with two recent systematic literature reviews, one of which focused on OUD patients’ perspectives on medication treatments [39] and the other on barriers to accessing opioid maintenance treatments [40]. Both systematic reviews found that patients had positive attitudes toward OUD treatments in which anonymity was guaranteed, and the latter revealed that patients were worried during treatment that their anonymity would be broken. In the latter systematic review, OUD patients also reported stigma as one of the greatest barriers to opioid substitution therapy.

In our study, many patients expressed that stigma was a barrier to treatment and that they did not usually choose treatment programs that were stigmatized by people without OUD. Amini-Rarani et al. (2020) found that patients with OUD in Iran were discouraged from seeking treatment due to the indignity they felt as drug users. Other studies on patients with OUD conducted in diverse settings also reported stigma as a main barrier to entering/staying in treatment [41–45]. Thus, if treatment can bolster patients’ dignity, this might increase their intention to undergo and commitment to treatment.

Cioe et al. (2020) showed that some OUD patients chose special treatment programs because they perceived them as helpful in managing their withdrawal symptoms. Fear of withdrawal has been shown to be an important factor in the decision to undergo treatment [39]. Our study found that patients who reported that a program had minimal side effects and a positive effect on their quality of life were more satisfied with the treatment.

Nayak et al. (2021) highlighted the importance of considering the preferences of patients’ family members regarding OUD treatment options. However, the study focused only on familial perceptions of OUD treatments and not on patients’ perceptions [46]. Our study, instead tried to explore the preferences of patients. Khazaee-Pool et al. (2018) indicated that OUD patients engaging in MMT were more motivated to comply with treatment if their family members had a positive perception of the medication for OUD treatment. Swartz et al. (2022) further reported the influential role played by family members.

Our study also revealed that treatment costs, location of the treatment center, length of treatment, and frequency of attendance influenced treatment-seeking preferences. In addition, informed treatment and knowledgeable staff from a variety of specialties were favored. Costs and financial difficulties were repeatedly mentioned as prominent factors that resulted in negative attitudes toward treatment. Interestingly, a review of barriers to accessing opioid substitution treatment for OUD found that qualitative studies did not report the cost of treatment as a barrier as frequently as quantitative studies [40]. It seems that more research is needed to understand the role of financial costs in adherence to treatment.

Our study also identified that the location of the treatment center was important to patients. While some patients preferred a treatment center further away from the city center to maintain their anonymity, others were willing to choose a center close to their homes and workplaces. The preferences of the latter group of participants were consistent with studies showing that the distance of treatment centers, mostly medication centers, is a barrier for treatment continuance [40]. In addition, consistent with our study, frequent visits to treatment centers, especially for medication, is difficult to integrate into regular daily activities such as work [39, 40]. The more flexible OUD treatment is, the fewer negative perceptions patients tend to have [40, 47].

The results of our study suggest that treatment types, including maintenance vs. abstinence treatment, residential vs. nonresidential treatment, and community (group) vs. individual treatments, have their own strengths and weaknesses, which may serve as drivers of or threats to OUD treatment and relapse prevention. Addressing these strengths and weaknesses can be seen as a policy approach to encouraging opioid treatment and recovery. In our study, we found that while many patients perceived nonresidential treatments as a better fit for their personal lives and job conditions than residential options, others believed that residential treatment would help them avoid the triggers of relapse. Substance use treatment is a long-term process, and residential treatments usually include detoxification and should therefore be followed by subsequent abstinence/maintenance treatments in communities or individually.

Existing studies have indicated that some patients view maintenance treatments for OUD as replacing one drug with another. Furthermore, they have concerns about the difficulty of ending medications or believe that the side effects of available medications for OUD treatment are worse than illegal opioids [39, 40, 48]. Consistent with our study, it has also been shown that some patients with prior experience with abstinence programs believe that participating in MMT programs can lead to isolation and judgment. Furthermore, some patients believe that OUD patients in nonmedical treatment look down on those who choose maintenance treatment programs [39].

Although the literature on community (or group) treatments for OUD is limited [49], there is at least some evidence that these treatments are effective. For instance, a qualitative study revealed that weekly group visits helped OUD patients by offering emotional support and lightening their mood, and they expressed feelings of gratitude to the group. Weekly group visits foster a sense of accountability, a shared identity, and a supportive community, which are unlikely to be achieved through individual treatment [49]. In a study by Tuten et al. (2007), some patients reported that access to anonymous meetings was important for aftercare services, which is in line with the preferences of the patients in our study.

Thus, our findings revealed that patients with OUD compare the positive and negative aspects of different treatment programs and consider a set of qualities and a combination of decisions when choosing treatment options. Our findings may inform policymakers about which aspects of OUD treatment programs are preferred by patients. Providing treatment programs that conform to patients’ preferences may enhance treatment-seeking behaviors and may also help motivate them to complete these programs. As described by Coffa and Snyder (2019), patient preference is an important consideration in choosing the correct medication for OUD [45, 50, 51].

Our study has some limitations. First, our study was performed in one city in Iran, Isfahan, so the findings cannot be generalized to other contexts. We aimed to explain treatment preferences in depth and present the various viewpoints of OUD patients rather than striving for a singular truth or generalization [52]. Therefore, we applied a constructionist approach [53], which limits generalizability. Second, although confidentiality and privacy were emphasized throughout the interviews, the answers provided by some study participants may still have been biased, and some OUD patients may not have told the full truth or articulated their desires, instead narrating what they thought the interviewers expected or wanted to hear.

Finally, this study focused only on patients’ preferences. Although considering patients’ preferences may improve the outcomes of evidence-based medicine, inconsistencies may exist between evidence-based medicine and what patients want. More research is needed to ensure that both can be integrated in the best possible way.

## Conclusions

The study identified three aspects of treatment preferences—treatment concerns, treatment attributes, and treatment types—that affect treatment-seeking behaviors. OUD patients assess each treatment program using a set of qualities, and they have different preferences for treatment programs. The identified themes could inform policymakers about the treatment preferences of male patients and provide an opportunity to promote better treatment options for OUD.

## Electronic supplementary material

Below is the link to the electronic supplementary material.

Supplementary Material 1

Supplementary Material 2

## Data Availability

The datasets generated and/or analyzed during the current study are not publicly available due to participants’ anonymity, but are available from the corresponding author on reasonable request.

## List of abbreviations

OUD: Opioid use disorder
MMT: Methadone maintenance treatment
DICs: Drop-in centers

## References

[1] Merz F (2018). United Nations Office on Drugs and Crime: World Drug Report 2017. 2017. SIRIUS–Zeitschrift für Strategische Analysen.

[2] Alam-mehrjerdi Z, Noori R, Dolan K (2016). Opioid use, treatment and harm reduction services: the first report from the Persian Gulf region. J Subst Use.

[3] Razzaghi E, et al. Rapid Situation Assessment (RSA) of drug abuse in Iran. Prevention Department, State Welfare Organization, Ministry of Health, IR of Iran and United Nations International Drug Control Program,; 1999.

[4] Amin-Esmaeili M (2016). Epidemiology of illicit drug use disorders in Iran: prevalence, correlates, comorbidity and service utilization results from the iranian. Mental Health Survey Addiction.

[5] Alammehrjerdi Z et al. *Therapeutic Community Program for opioid-dependent treatment seekers*. Iran J Psychiatry Behav Sci, 2017. 11(3).

[6] Pashaei T (2014). Predictors of treatment retention in a major methadone maintenance treatment program in Iran: a survival analysis. J Res health Sci.

[7] Pashaei T (2013). Effectiveness of relapse prevention cognitive-behavioral model in opioid-dependent patients participating in the methadone maintenance treatment in Iran. Iran J public health.

[8] Alam-mehrjerdi Z (2015). Drug use treatment and harm reduction programs in Iran: a unique model of health in the most populated Persian Gulf country. Asian J Psychiatry.

[9] Rahimi-Movaghar A, et al. Assessment of situation and response to drug use and its harms in the Middle East and North Africa, 2012. Middle East and North Africa Harm Rediction Association (MENAHRA); 2013.

[10] Babaie E, Razeghi N (2013). Comparing the effects of methadone maintenance treatment, therapeutic community, and residential rehabilitation on quality of life and mental health of drug addicts. Addict health.

[11] Lawrinson P (2008). Key findings from the WHO collaborative study on substitution therapy for opioid dependence and HIV/AIDS. Addiction.

[12] Ahmadi J (2002). Buprenorphine maintenance treatment of heroin dependence: the first experience from Iran. J Subst Abuse Treat.

[13] Shirinbayan P (2010). Predictors of retention in methadone maintenance therapy: a prospective multi-center study. Sci Res Essays.

[14] Alavian SM et al. *Effectiveness of methadone maintenance treatment in prevention of hepatitis C virus transmission among injecting drug users*. Hepat monthly, 2013. 13(8). 10.5812/hepatmon.12411 PMC3782738 24069039

[15] Noori R (2012). Methadone maintenance therapy outcomes in Iran. Subst Use Misuse.

[16] Marshall T (2018). Patient engagement, treatment preferences and shared decision-making in the treatment of opioid use disorder in adults: a scoping review protocol. BMJ open.

[17] Friedrichs A (2016). Patient preferences and shared decision making in the treatment of substance use disorders: a systematic review of the literature. PLoS ONE.

[18] Pirnia B (2020). COVID-19 pandemic and addiction: current problems in Iran. Asian J psychiatry.

[19] Khazaee-Pool M et al. *Perceived barriers to methadone maintenance treatment among iranian opioid users*. Int J Equity Health, 2018. 17(1). 10.1186/s12939-018-0787-z PMC5996552 29890990

[20] Barry MJ, Edgman-Levitan S (2012). Shared decision making—the pinnacle of patient-centered care. N Engl J Med.

[21] Keirns CC, Goold SD (2009). Patient-centered care and preference-sensitive decision making. JAMA.

[22] Shay LA, Lafata JE (2015). Where is the evidence? A systematic review of shared decision making and patient outcomes. Med Decis Making.

[23] Montori VM, Gafni A, Charles C (2006). A shared treatment decision-making approach between patients with chronic conditions and their clinicians: the case of diabetes. Health Expect.

[24] Winstanley EL (2014). Improving treatment for opioid dependence: a perspective from the Ohio Valley node of the NIDA clinical trials network. Progress in community health partnerships: research education and action.

[25] Hohmann LA et al. *Comparative effectiveness of opioid abuse treatments: a systematic review*. Value in Health: The Journal of the International Society for Pharmacoeconomics and Outcomes Research, 2017. 20(5).

[26] Williams AR (2018). Developing an opioid use disorder treatment cascade: a review of quality measures. J Subst Abuse Treat.

[27] Sokol R (2018). Group-based treatment of opioid use disorder with buprenorphine: a systematic review. J Subst Abuse Treat.

[28] Ludwig AS, Peters RH (2014). Medication-assisted treatment for opioid use disorders in correctional settings: an ethics review. Int J Drug Policy.

[29] Marshall T et al. *Patient engagement, treatment preferences and shared decision-making in the treatment of opioid use disorder in adults: a scoping review protocol*. BMJ Open, 2018. 8(10). 10.1136/bmjopen-2018-022267 PMC6196846 30337310

[30] Toce MS (2018). Pharmacologic treatment of opioid use disorder: a review of Pharmacotherapy, Adjuncts, and toxicity. J Med Toxicol.

[31] Volkow NDMD (2019). Prevention and Treatment of Opioid Misuse and Addiction: a review. JAMA Psychiatry.

[32] Korthuis P (2017). Primary care-based models for the treatment of opioid use disorder: a scoping review. Ann Intern Med.

[33] Chetty M, et al. A systematic review of health economic models of opioid agonist therapies in maintenance treatment of non-prescription opioid dependence. Addiction Science & Clinical Practice; 2017. p. 12. 10.1186/s13722-017-0071-3 PMC5324212 28235415

[34] Ling S (2019). A systematic review of sex differences in treatment outcomes among people with opioid use disorder receiving buprenorphine maintenance versus other treatment conditions. Drug Alcohol Depend.

[35] Carew AM, Comiskey C (2018). Treatment for opioid use and outcomes in older adults: a systematic literature review. Drug Alcohol Depend.

[36] Tubog TDDC (2019). Use of Nalbuphine for treatment of Neuraxial Opioid-Induced Pruritus: a systematic review and Meta-analysis. AANA J.

[37] Kallio H (2016). Systematic methodological review: developing a framework for a qualitative semi-structured interview guide. J Adv Nurs.

[38] Fereday J, Muir-Cochrane E (2006). Demonstrating rigor using thematic analysis: a hybrid approach of inductive and deductive coding and theme development. Int J qualitative methods.

[39] Cioe K et al. *A systematic review of patients’ and providers’ perspectives of medications for treatment of opioid use disorder*. J Subst Abuse Treat, 2020: p. 108146. 10.1016/j.jsat.2020.108146 PMC7609980 33138929

[40] Hall NY et al. *Barriers to accessing opioid substitution treatment for Opioid Use Disorder: A systematic review from the client perspective* Drug and alcohol dependence, 2021: p. 108651. 10.1016/j.drugalcdep.2021.108651 33667783

[41] Hall NY et al. *Identifying the most common barriers to opioid agonist treatment in an australian setting*. Aust J Prim Health, 2023. 10.1071/PY22269 36934460

[42] Steiro A, Hestevik CH, Muller AL. *Patient’s and healthcare provider’s experiences with Opioid Maintenance Treatment (OMT): A qualitative evidence synthesis* 2023. 10.1186/s12913-024-10778-7 PMC10938774 38481254

[43] Husain JM (2023). A qualitative analysis of barriers to opioid agonist treatment for racial/ethnic minoritized populations. J Subst Abuse Treat.

[44] Swartz N (2022). Sick and tired of being sick and tired”: exploring initiation of medications for opioid use disorder among people experiencing homelessness. J Subst Abuse Treat.

[45] Kaplowitz E (2022). Treatment preference for opioid use disorder among people who are incarcerated. J Subst Abuse Treat.

[46] Nayak SM (2021). Familial perceptions of appropriate treatment types and goals for a family member who has opioid use disorder. Drug Alcohol Depend.

[47] Checkley L, Steiger S, Knight KR (2022). I wanted something that was more flexible”: a qualitative study of patient preferences on choosing buprenorphine over methadone in a large, safety-net hospital opioid treatment program. Substance abuse.

[48] Wyse JJ et al. *“I’m Clean and Sober, But Not Necessarily Free”: Perceptions of Buprenorphine Among Patients in Long-Term Treatment* Substance Abuse, 2023: p. 08897077231165625. 10.1177/08897077231165625 PMC11132627 37226910

[49] Sokol R et al. *Building a Group-Based Opioid Treatment (GBOT) blueprint: a qualitative study delineating GBOT implementation* Addiction science & clinical practice, 2019. 14(1): p. 1–17. 10.1186/s13722-019-0176-y PMC6935085 31882001

[50] Coffa D, Snyder H (2019). Opioid use disorder: medical treatment options. Am Family Phys.

[51] Puglisi LB (2019). Medications for opioid use disorder among incarcerated individuals: a review of the literature and focus on patient preference. Curr Addict Rep.

[52] Bengtsson M (2016). How to plan and perform a qualitative study using content analysis. NursingPlus Open.

[53] Bryman A. Social research methods. Oxford university press; 2016.

