# Supplementary material for: Male patients’ preferences for opioid use treatment programs
Source: BMC Psychiatry. 2023 Jun 16;23:440. doi: 10.1186/s12888-023-04939-x (PMC10273501; doi:10.1186/s12888-023-04939-x)
Supplement: Supplementary file 1 — Supplementary Material 1 [file 12888_2023_4939_MOESM1_ESM.docx]

**Detailed characteristics of all participants**

| **Participant number** | **Age** | **Addiction duration** | **Education level** | **Marital status** |
| --- | --- | --- | --- | --- |
| 1 | 49 | 30 | High school | Married |
| 2 | 45 | 26 | Primary | Married |
| 3 | 37 | 14 | Secondary | Single |
| 4 | 38 | 18 | Secondary | Single |
| 5 | 23 | 3 | Illiterate | Single |
| 6 | 35 | 18 | High school | Married |
| 7 | 30 | 9 | High school | Married |
| 8 | 33 | 2 | Primary | Married |
| 9 | 32 | 9 | Secondary | Married |
| 10 | 31 | 10 | High school | Single |
| 11 | 45 | 20 | Primary | Single |
| 12 | 36 | 15 | Secondary | Single |
| 13 | 32 | 15 | Primary | Married |
| 14 | 39 | 20 | High school | Single |
| 15 | 43 | 17 | High school | Married |
| 16 | 57 | 10 | Primary | Married |
| 17 | 54 | 20 | High school | Single |
| 18 | 42 | 13 | Primary | Married |
| 19 | 41 | 1 | High school | Married |
| 20 | 54 | 5 | Academic | Married |
| 21 | 33 | 6 | High school | Married |
| 22 | 42 | 10 | High school | Married |
| 23 | 60 | 10 | Academic | Married |
| 24 | 48 | 15 | Secondary | Married |
| 25 | 26 | 10 | High school | Single |
| 26 | 24 | 5 | High school | Single |
| 27 | 26 | 5 | Academic | Single |
| 28 | 24 | 8 | High school | Single |
| 29 | 36 | 20 | Secondary | Married |
| 30 | 46 | 10 | Academic | Married |
| 31 | 39 | 14 | High school | Single |
| 32 | 25 | 11 | Academic | Single |
| 33 | 24 | 3 | High school | Single |
| 34 | 34 | 17 | High school | Single |
| 35 | 28 | 10 | Secondary | Married |
| 36 | 33 | 13 | Secondary | Single |
| 37 | 30 | 4 | High school | Married |
| 38 | 51 | 25 | Secondary | Married |
| 39 | 30 | 8 | High school | Married |
| 40 | 32 | 10 | Academic | Married |
| 41 | 27 | 4 | Academic | Single |
| 42 | 37 | 10 | High school | Single |
| 43 | 34 | 10 | Primary | Married |
| 44 | 37 | 25 | Secondary | Married |
| 45 | 34 | 5 | Academic | Single |
| 46 | 62 | 32 | Illiterate | Married |
| 47 | 26 | 1 | Secondary | Single |
| 48 | 31 | 10 | Secondary | Single |
| 49 | 40 | 3 | Primary | Married |
| 50 | 30 | 11 | Academic | Single |
| 51 | 25 | 9 | High school | Married |
| 52 | 34 | 8 | Primary | Married |
| 53 | 30 | 2 | Secondary | Married |
| 54 | 30 | 5 | Academic | Married |
| 55 | 24 | 3 | High school | Married |
| 56 | 36 | 10 | High school | Married |
| 57 | 33 | 17 | Academic | Single |
| 58 | 49 | 25 | High school | Married |
| 59 | 40 | 22 | Primary | Single |
| 60 | 43 | 12 | Secondary | Divorced |
| 61 | 33 | 18 | Primary | Single |
| 62 | 55 | 13 | Primary | Divorced |
| 63 | 55 | 20 | Primary | Married |
| 64 | 42 | 17 | Secondary | Single |
