# Supplementary material for: Male patients’ preferences for opioid use treatment programs
Source: BMC Psychiatry. 2023 Jun 16;23:440. doi: 10.1186/s12888-023-04939-x (PMC10273501; doi:10.1186/s12888-023-04939-x)
Supplement: Supplementary file 2 — Supplementary Material 2 [file 12888_2023_4939_MOESM2_ESM.docx]

**Interview Guide: Male client preferences of opioid use treatment attributes**

- Demographic Characteristics

1. Age
2. Job (at the time of interview)
3. Education history
4. Marriage status (at the time of interview)

- Drug use History
  1. Years of regular opioid use
  2. Types of opioids used regularly
  3. Experience of drug use injection
  4. History of opioid treatment

| **Focus area** | **Example of questions and probes** |
| --- | --- |
| cost of OUD treatment | - Describe your preference over treatment cost  - Describe financial burdens of treatment for you/your family  - Example of affordable treatment program(s) |
| Environment of treatment centers | - What does accessible treatment center mean to you/your family?  - Describe your preference over the location of treatment centers  - Describe your preference over your relationship with other opioid patients in treatment centers  - Describe your preference over skills , professions and communication of therapists/treatment provider |
| Family support | - Describe how family’s preference over treatment program may influence your treatment choice?  - Describe how do you fell about the role of your family during treatment process? |
| Treatment Duration | - Describe your preference over duration of treatment  - Describe your preference over duration between therapy visits |
| Residential/Non-residential treatment | - Describe your reasons behind your preference over Residential/Non-residential treatment programs |
| Maintenance/Abstinence treatment | - Describe your reasons behind your preference over Maintenance/Abstinence treatment |
| Patients’ Perception | - How could a OUD treatment program be improved  - How good/bad experiences have you had during current/previous treatment attempts?  - Describe your incentives for choosing current treatment option  - Describe your concern about the current treatment option |
